# Recruitment and Early Retention of Women with Advanced Breast Cancer in a Complementary and Alternative Medicine Trial

**DOI:** 10.1093/ecam/nep051

**Published:** 2011-02-14

**Authors:** Alla Sikorskii, Gwen K. Wyatt, Azfar-e-Alam Siddiqi, Deimante Tamkus

**Affiliations:** ^1^Department of Statistics and Probability, College of Natural Science, Michigan State University, East Lansing, MI 48824, USA; ^2^College of Nursing, Michigan State University, East Lansing, MI 48824, USA; ^3^College of Human Medicine, Michigan State University, East Lansing, MI 48824, USA

## Abstract

More than 80% of women with breast cancer are now reported to be using complementary and alternative medicine (CAM) therapies during conventional treatment. A randomized clinical trial (RCT) of reflexology with late stage breast cancer patients serves as the data source for this article. The purposes were to investigate: (i) reasons for refusal to participate in a RCT of reflexology; (ii) the differences between those who completed the baseline interview and those who dropped out before baseline; and (iii) the utility of the Palliative Prognostic Score (PPS) as a prognostic screening tool in minimizing early attrition (before baseline) from the trial. Eligible women (*N* = 400) approached at 12 cancer centers in the Midwest had advanced breast cancer, were on chemotherapy or hormonal therapy, and had a PPS of 11 or less. Comparisons of those who dropped out early (*N* = 33) to those who stayed in the trial (*N* = 240) were carried out using Wilcoxon rank, *t*-, chi-squared and Fisher's exact tests. The reasons of being “too sick” or “overwhelmed” were given by less than 12% of the women who refused to participate. There was a higher early dropout rate among black women compared to other (primarily white) women (*P* = .01). Cancer recurrence and metastasis, age, and the PPS were not predictive of early retention of women. Specialized techniques may be needed to ensure black women remain in the trial once consented. Women with advanced disease were likely to enter and remain in the trial despite deterioration in health.

## 1. Introduction

Breast cancer is the leading malignancy and the second cause of cancer-related deaths among women [1]. Symptoms related to cancer and its treatment persist for long periods after diagnosis and treatment and can worsen as the disease becomes more advanced [2–4]. Symptoms are the strongest predictors of patients' overall quality of life (QOL), particularly among those nearing the end of life [5]. Understandably, many women with advanced breast cancer are turning to complementary and alternative medicine (CAM) therapies as supportive care during cancer treatment [6–8]. Over the last few decades CAM therapies have gained popularity, and according to a 2007 survey, as many as 80% of women diagnosed with breast cancer report using CAM for symptom management and improving their QOL [9].

The challenge to investigating popular CAM therapies among cancer patients with advanced disease can be recruitment and retention in a randomized clinical trial (RCT) [10, 11]. With some herbal therapies, recruitment into RCT may be the greatest challenge since patients who agree to participate and be randomized may be different from those who refuse [12]. Although there is some debate over how best to study the efficacy of CAM therapies [12, 13], the RCT nevertheless remains the gold standard.

The accuracy and usefulness of the findings from a RCT of a CAM therapy, like any other clinical trial, are only as good as the internal and external validities of the trial itself. Internal validity refers to the validity of inferences drawn from a study [14]. External validity, on the other hand, reflects the extent to which the findings from the study can be generalized. Most important in ensuring the internal and external validity is the selection of a study sample representative of the target population [15, 16].

Little is known about the characteristics of non-participants or early dropouts (those who drop out before baseline interview, which constitutes the formal initiation of the trial). The data on those who refuse to participate are rarely available due to human subject research guidelines, and therefore this assessment of non-participants is restricted to a description of reasons for refusal to participate. After consenting to participate in a CAM therapy RCT, some women with advanced breast cancer may drop out for various reasons related to deterioration in health, exacerbation of symptoms, or death. A high attrition rate leads to two concerns: (i) patients who drop out are not exposed to the intervention and receive no benefit, and (ii) the internal and external validities of the trial can be compromised [17, 18] because, due to attrition, the sample may not be representative of the target population or of the population of those who can potentially benefit from the intervention.

A review of the pertinent literature did not reveal any published research addressing recruitment and attrition issues in CAM therapy trials. A review of RCTs of Tai Chi conducted between 1966 and 2007, for example, indicated lack of adequate reporting of several elements of the trial design and analysis, specifically lack of a clear description of recruitment and absence of attrition analyses [19]. Findings on recruitment reported from other trials are summarized in an exemplary review by Lovato et al. [20]. Minorities and women tend to be more difficult to recruit in controlled trials than the majority and male participants. However, since the issuance of National Institutes of Health (NIH) guidelines for targeted recruitment of women and minorities, some increase in successful recruitment has been documented [21].

Factors associated with attrition among non-CAM studies demonstrate substantial variation, not only in the kind of disease being studied but also by socio-demographic and clinical characteristics of the participants. In several studies, attrition was higher among patients of low socioeconomic status (SES), with low education, and among racial minorities [22–24]. Age of patients is commonly reported as a predictor of attrition; however, the direction of the effect of age is less consistent. Several studies report older patients to be more likely to drop out, whereas others reported younger patients to be at greater risk of leaving [25, 26].

Literature suggests that patients' characteristics such as functional limitations, poor self-reported health, unstable health status, cognitive impairment, and major disease, particularly depression, are found to be significantly associated with attrition [27–32]. In their research, Mihelic et al. [33] reported finding sicker patients with a high number of comorbidities and higher level of illness severity to be more likely to drop out than those with better health statuses. Conversely, Bender et al. found the opposite: Patients with fewer coexisting chronic conditions and lower illness severity were more likely to drop out compared to sicker patients [22]. These conflicting findings are likely to be the result of the different nature of interventions and target populations in these two studies. Therefore, investigation of factors related to attrition in CAM therapy trials among patients with advanced disease is warranted, and cancer patients nearing end of life may be a group with unique dynamics, making it distinctive from other groups of patients.

In trials with cancer patients nearing end of life, one of the leading reasons for attrition may be deterioration in health or death. On the other hand, patients with high symptom burden may benefit from CAM supportive care intervention. Due to attrition, typically only baseline data are available for these patients; these data cannot be used in rigorous testing of the efficacy of CAM intervention without making certain assumptions; for example, the assumption of data missing at random. The assumptions underlying analyses with missing data are not always verifiable in practice [34]. Therefore, exclusion of patients who are highly unlikely to survive through the duration of trial may be necessary to avoid relying on unverifiable assumptions in efficacy testing. A commonly used scale for predicting survival is the Palliative Prognostic Score (PPS) [35, 36].

It was developed using a sample of 519 hospice-home care patients, and validated in the populations of patients with terminal cancer and advanced cancer [37–39]. However, it is unknown whether better survival probability is associated with retention in CAM clinical trials.

This article addresses the authors' current use of the PPS as one of the inclusion criteria in an ongoing trial of reflexology, a commonly used CAM therapy, among women with advanced breast cancer. To date, the study has 277 women consented with the target recruitment of 390. This report is guided by the following research questions, among women with advanced breast cancer:

- What are the most frequent reasons for refusal to participate in a CAM RCT of reflexology?
- Among those who consent, what are the differences between those who completed the baseline interview and those who dropped out before baseline?
- What is the utility of the PPS as a prognostic screening tool in minimizing attrition from the CAM RCT of reflexology?

We focus on early attrition, that is, attrition before baseline interview and randomization. Factors that influence attrition before and after randomization may be different, as attrition post-randomization may be due to reasons related to the intervention protocol or allocation into control group [40].

## 2. Methods

The study was approved by the Institutional Review Board of the University and those of each of the 12 participating medical oncology settings in the Midwest. The sites represented the standard of care for oncology in the Midwest and comprised a combination of free-standing cancer treatment facilities and one comprehensive cancer center.

**Figure 1:**
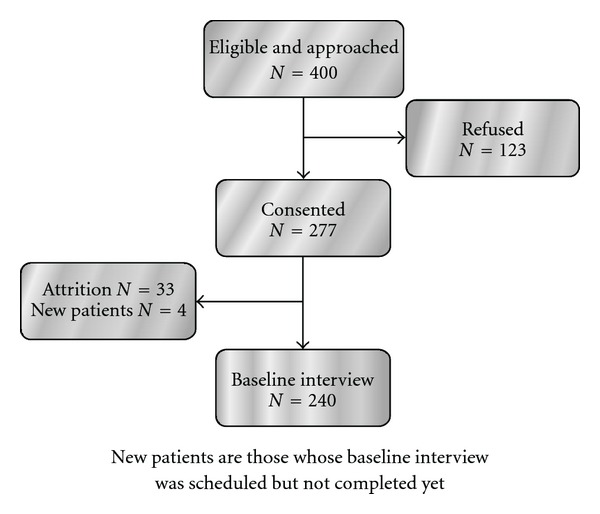
Flowchart of the study.

Eligibility criteria included: (i) being a woman 21 years of age or older; (ii) having diagnosis of stage III or IV breast cancer, or initial diagnosis of stage I or II with a later recurrence or metastasis; (iii) being able to perform basic activities of daily living; (iv) being cognitively intact and free of a charted diagnosis of mental illness (the recruiter reviewed the chart for diagnosis of mental illness and asked women three cognitive orientation questions, on time, place, and person); (v) being able to speak and understand the English language; (vi) having access to a telephone; (vii) being able to hear normal conversation; (viii) receiving chemotherapy or hormonal therapy at intake into the study; and (ix) having a score of 11 or lower on the Palliative Prognostic Score scale. Exclusion criteria for patients to enter the study included: (i) receiving hospice care at intake; (ii) residing in a nursing home or similar care facility; (iii) being bedridden; (iv) regularly using complementary therapies similar to those used in the protocol (e.g., reflexology, foot massage, pedicure with massage); (v) participating in a new drug experimental chemotherapy; and (vi) undergoing bone marrow transplantation. Nurses employed at the recruitment sites were trained to implement the recruitment protocol. Eligible women were approached, given an explanation of the trial, and invited to sign an informed consent. For those who refused to participate, nurses obtained reasons for refusal. Following consent, enrollment data were collected from the medical record and a baseline telephone interview was scheduled. For early dropouts (those who consented but did not complete baseline interview), attrition reasons were recorded. Upon completion of baseline interview, women were randomly allocated to one of the following three groups: (i) intervention (foot reflexology); (ii) placebo foot treatment; or (iii) control. Women in all three groups continued to receive standard medical care. The participants randomized to reflexology or placebo foot sessions received intervention sessions either at home or at the oncology clinic. In the latter case, sessions were scheduled at participants' convenience and were coordinated with the times women came to clinic for their appointments. This research is based on data collected before baseline interview and randomization. These data were collected from medical records and are limited by the availability of the information to demographic and clinical variables.  Figure 1 summarizes the numbers of women who were eligible and approached, refused, consented, and those who dropped out before baseline interview. Because the study is still in progress, all women who were enrolled but whose baseline was pending were not included in the analysis.

### 2.1. Measures

Nurse recruiters recorded reasons stated by those who refused to participate. For those who consented, we used measures from the medical record in regard to evaluating attrition.

#### 2.1.1. Demographics

These data included age, race, ethnicity, marital status and employment.

#### 2.1.2. Disease Characteristics

The medical record was also the source for data on cancer stage, recurrence and metastasis, and goal of therapy (curative, palliative, maintenance, or other).

#### 2.1.3. Palliative Prognostic Score

The PPS determined the probability of an advanced cancer patient's survival over the next 30 days. The PPS score is based on six factors identified as predictive of survival in terminally ill cancer patients and includes: Clinical Prediction of Survival (CPS), Karnofsky Performance Status (KPS) [41], anorexia, dyspnea, total white blood cell count (WBC), and lymphocyte percentage. Each variable is assigned a numeric score and a sum of the scores is calculated for each patient, which can then be used to assign patients to one of three risk groups. The scores potentially range from 0 to 17.5, where a higher score represents poor performance. A score less than or equal to 5.5 indicates a 70% chance of surviving for 30 days. If the score is between 5.6 and 11.0, the chance of survival is 30%–70%, while a score greater than 11.0 indicates a probability less than 30% [16]. Since a cutoff of 11 was one of the inclusion criteria, all patients in this study had a PPS of 11 or less.

Among those who consented, attrition was defined by the completion status of the baseline interview. After excluding pending patients, all patients who had not completed baseline interview for any reason were considered early dropouts.

### 2.2. Data Analyses

For those who did not consent, refusal reasons were tabulated. Among those who consented, comparisons of those retained and early dropouts were carried out on demographics, cancer recurrence and metastasis, and the PPS. Categorical variables were compared using chi-square or Fisher's exact tests. Race categories were grouped as black versus other (primarily white with a few Asian, American Indian, or Alaskan women). Marital status was grouped as married or living with a partner versus not. *T*-tests were used for comparisons of variables that were approximately normally distributed (e.g., age). The Wilcoxon rank test was performed to compare the distribution of PPS for early dropouts and those who remained in the trial up to baseline interview. All analyses were performed using SAS 9.1 software [42].

## 3. Results

**Research Question 1**

What are the most frequent reasons for refusal to participate in a CAM RCT of reflexology?

Women's stated reasons for refusal to participate in the study are listed in Table 1. Despite advanced disease, the reasons of being “too sick" or “overwhelmed" were given by less than 12% of the women who refused to participate. Disinterest in research, explicitly stated or implicit (no reason given) accounted for 40% of refusals.

**Research Question 2**

Among those who consent, what are the differences between those who completed the baseline interview and those who dropped out before baseline?

Table 2 summarizes the characteristics of those who consented but dropped out before baseline interview, and those who completed baseline interview. Even though the majority of the sample was white, reflective of the population of patients treated at the recruitment sites, there was a significantly higher early dropout rate among black women compared to other (23.5% versus 10.5%, *P* = .01). No differences between early dropouts and those retained up to the baseline interview were found on age, employment, cancer recurrence, metastasis, and goal of therapy. The early dropout rate among women who were married or living with a partner was 9.5% versus a 16.9% among those who were never married, divorced, or separated. However, with available sample size this difference was not statistically significant (*P* = .08).

In this study breast cancer patients were treated either with chemotherapy or hormonal therapy. Among early dropouts, 63% received chemotherapy and 37% received hormonal therapy. In general, chemotherapy is associated with more acute toxicity than hormonal therapy, which could lead to higher dropout rate. Due to differences seen in dropouts among black women compared to non-black women, we evaluated the association between treatment and race among early dropouts. Chemotherapy was administered for 57% of black patients as compared to 65% non-black patients (Fisher's exact *P*-value = .99, not in tables). Therefore, the higher rate of dropouts seen in black women cannot be explained by treatment administered.

Of the 33 early dropouts, 10 (30%) were too sick or died, seven (21%) were too busy and two women stated other unique personal reasons for attrition. The remaining 42% of early dropouts changed their mind about participation or could not be reached via the telephone.

**Research Question 3**

What is the utility of the PPS as a prognostic screening tool in minimizing attrition from the CAM RCT of reflexology?

When early dropouts were compared to those who completed baseline interview on PPS, no differences were found either in the comparisons of the means or of the distributions (Table 2). Thus the PPS in the range of 11 or below has not been found to be predictive of retention in the reflexology trial with advanced breast cancer women.

## 4. Discussion

This research addressed two issues essential to securing external validity for a trial of a CAM therapy directed at improving QOL for women with advanced breast cancer. First is the assessment of reasons given by women who refused to participate in a CAM trial or who dropped out shortly after giving consent and before beginning the trial. This initial assessment provided information about those who did not participate in the trial and to whom the findings cannot be generalized. Second, those who remain in the trial were compared to those who drop out early on several characteristics including the PPS. Such comparisons further shed light on the generalizability of findings beyond the sample of those who complete the study.

Women with advanced breast cancer were likely to enter the trial, with *∼*70% of those approached agreeing to participate. These findings are consistent with Boon et al. [9] who found that more than 80% of women with breast cancer used CAM therapies and therefore could be interested in participating in a CAM trial. These findings are an indication of women's interest in supportive care they may receive from using the CAM therapy reflexology for symptom management during chemotherapy. The high rate of consent also suggests the external validity of findings that can be generalized to the population of women with late stage breast cancer.

Those who refused to participate were not sicker patients, but rather those who were not interested in research in general or in reflexology specifically. The refusals due to strong preferences that are common in the context of CAM are reported in the literature [12], however reasons of not agreeing to be randomized was not stated by women in this trial. A very small percentage of refusals were due to being too sick. This finding supports the external validity of trial results because women who could potentially benefit from the intervention agreed to participate.

The finding of a larger dropout rate among black women is consistent with results of Fair and colleagues [43]. They successfully recruited a targeted number of black women to a noninvasive study of mammography, however black women were more likely to drop out of the study compared to their white counterparts with 58% of early dropouts being black. Clinical recruiters may need to implement specialized techniques to ensure black women remain in the trial once consented.

The use of the PPS of 11 or less as one of the inclusion criteria was informed by the literature suggesting that poor functional status may be predictive of attrition. Higher likelihood of dropping out for sicker patients with higher symptom burden was reported in cancer symptom management trials prior to randomization [40]. Therefore the finding of the PPS not being useful for prediction of early retention was somewhat unexpected. Karnofsky's performance status is one of the components of the PPS, and because more than 50% of palliative prognostic scores were zero, the KPS contributed zero to the PPS for more than half of the patients. However, the cut-off point for KPS contribution to the PPS is 30. A patient scored at 30 is severely disabled, requiring considerable assistance with activities of daily living and frequent medical care. Thus a patient may be clinically quite ill and yet have low PPS. Another limitation of the PPS is its reliance on clinician prediction of survival for approximately 50% of the total score. Reviews of studies have found that clinician estimates of survival are generally inaccurate and systematically over-optimistic [38, 44]. Even though the trial is still in progress and has not reached the final sample size, the finding that the PPS was not predictive of early attrition was not due to insufficient power to detect the difference, but because the difference in PPS was not observed. The effect size for the differences on PPS between those who dropped out and those who stayed was virtually zero, therefore even with larger sample size, the PPS would not be significantly associated with attrition.

Several limitations of this research should be noted. While information on additional factors that may be related to attrition such as socioeconomic status (SES), prior CAM use, and social support was collected in baseline interview, women who dropped out before the baseline interview did not have such data collected. Demographic data collected from medical records provide proxies for some of these variables, for example, marital status may reflect social support, and employment status may reflect SES. We report on attrition before randomization; therefore, we did not consider attrition by trial arm. All patients included had a PPS of 11 or less, thus, the utility of PPS across the range of 11.5–17.5 in minimizing attrition and securing external validity of the trial remains open to question. A relatively small proportion of black women remained in trial to complete baseline interview. It is unknown what the rate of consent was among different racial groups as race data was not available for those who did not consent. Additionally, very few Hispanic women were enrolled reflective of the ethnic distribution in the population treated at the recruitment locations.

CAM therapies have not been studied extensively with RCT designs [45, 46], and the findings on attrition from other symptom management trials may or may not apply to CAM trials. Furthermore, among published RCTs of CAM, quality of reporting is often not adequate, with insufficient information about recruitment and retention in particular [19, 47]. This article is among the first to reveal factors associated with patients' interest and decisions to remain in CAM therapy trials.

Knowledge about the reasons for refusal and attrition of advanced breast cancer patients in trials can inform inclusion criteria and retention strategies of future CAM trials. For example, because sicker patients may be attracted to CAM therapies for symptom management and remain in the trial despite deterioration in health, exclusions based on the PPS score of higher than 11 may not be warranted. Participant recruiters may successfully approach women with high symptom burden to offer participation in CAM trials since the intervention may be beneficial as supportive care during treatment.

## Figures and Tables

**Table 1:** List of reasons given for refusal to participate.

Reason for refusal to participate	*N* (%)
Too busy	31 (25.20)
No reason	27 (21.95)
Not interested	22 (17.89)
Foot concerns	10 (8.13)
Other	17 (13.82)
Too sick	6 (4.88)
Overwhelmed by prospect of research	8 (6.50)
Do not like being interviewed	2 (1.63)

**Table 2:** Comparison of characteristics of patients who consented but dropped out early to those who completed intake interview.

Characteristics	Early drop-outs *N* (%)	Completed baseline interview *N* (%)	*P*-value
Age^a^	58.12 (12.66)	57.34 (11.36)	.71
Palliative Score^a^	0.79 (1.09)	0.76 (1.10)	.88
Race			.01^b^
White	25 (75.76)	212 (88.70)	
Black	8 (24.24)	23 (9.62)	
American Indian or Alaskan Native	0 (0.00)	1 (0.42)	
Asian	0 (0.00)	2 (0.84)	
Refused	0 (0.00)	1 (0.42)	
Ethnicity			.41
Non-Hispanic	32 (96.97)	235 (98.33)	
Hispanic	1 (3.03)	3 (1.26)	
Refused	0 (0.00)	1 (0.42)	
Marital status			.08^c^
Married or living with a partner	17 (51.52)	162 (67.78)	
Divorced/separated	8 (24.24)	28 (11.72)	
Never married	3 (9.09)	27 (11.30)	
Widowed	4 (12.12)	19 (7.95)	
Refused	1 (3.03)	3 (1.26)	
Type of employment			.72^d^
Retired	9 (27.27)	59 (24.69)	
Part time	6 (18.18)	23 (9.62)	
Full time	7 (21.21)	64 (26.78)	
Disabled	3 (9.09)	29 (12.13)	
Not employed	3 (9.09)	37 (15.48)	
Homemaker	4 (12.12)	21 (8.79)	
Refused	1 (3.03)	6 (2.51)	
Palliative score			.92
0	19 (57.58)	131 (54.81)	
0.5	0 (0.00)	10 (4.18)	
1.0	4 (12.12)	47 (19.67)	
1.5	4 (12.12)	12 (5.02)	
2.0	2 (6.06)	8 (3.35)	
2.5	2 (6.06)	17 (7.11)	
3.0	1 (3.03)	4 (1.67)	
3.5	0 (0.00)	4 (1.67)	
4.0	1 (3.03)	4 (1.67)	
5.5	0 (0.00)	2 (0.84)	
Metastatic cancer			.56
Yes	23 (69.70)	178 (74.48)	
No	10 (30.30)	61 (25.52)	
Recurrent cancer			.36
Yes	13 (39.39)	75 (31.38)	
No	20 (60.61)	164 (68.62)	
Therapy goals			0.47
Maintenance	11 (33.33)	92 (38.49)	
Curative	9 (27.27)	78 (32.64)	
Palliative	6 (18.18)	41 (17.15)	
Uncertain	7 (21.21)	28 (11.72)	

^a^Mean and standard deviation.

^b^ *P*-value for the comparison between black versus other.

^c^ *P*-value for the comparison between those married or living with a partner versus other.

^d^ *P*-value for the comparison those employed versus those not employed.
